# Two new Cd(II)/Zn(II) coordination polymers: luminescence properties and synergistic treatment activity with ultrasound therapy on uterine fibroids

**DOI:** 10.1080/15685551.2022.2088976

**Published:** 2022-06-20

**Authors:** Hong-Mei Liu, Xiao-Na Shang

**Affiliations:** a Ultrasound Medicine Department, Sun Simiao Hospital of Beijing University of Traditional Chinese Medicine, Tongchuan, Shaanxi, China; b Ultrasound Department, Shaanxi Second Provincial People’s Hospital, Xi’an, Shaanxi, China

**Keywords:** Self-assembly reaction, blue luminescence, coordination polymer, uterine fibroids, ultrasound therapy

## Abstract

Through the self-assembly reaction of 5-substituted isophthalic acid and bis(imidazolyl) ligands with Cd(II) ion or Zn(II) ion, two new coordination polymers with the chemical formulae of [Cd(5-meo-ip)(bmip)]_n_ (**1**) and [Zn(5-pro-ip)(bip)]_n_·2 n(H_2_O) (**2**) (5-meo-H_2_ip = 5-methoxyisophthalic acid, 5-pro-H_2_ip = 5-propoxyisophthalic acid, bmip = 1,3-bis(2-methylimidazolyl)propane bip = 1,3-bis(imidazolyl)propane) were successfully obtained and structurally characterized by a series of characterization techniques. Moreover, compounds **1**–**2** show intense blue luminescence at room temperature. Furthermore, the assessment of their treatment activity on the uterine fibroids combined with ultrasound therapy was evaluated and the specific mechanism was investigated at the same time. Firstly, the effect of compound treatment on uterine fibroids apoptosis was detected via flow cytometry. Next, the apoptotic signaling pathway activation was detected through the Caspase-3 and Caspase-8 Activity Assay Kit.

## Introduction

The incidence of uterine fibroids ranks first among benign tumors of female reproductive organs. It is generally believed that the incidence of uterine fibroids is 5% to 50%, which can be as high as 70% [1]. It is reported in the literature that the youngest patient with uterine fibroids in foreign countries is 10 years old, while in China it is 15 years old, which is related to the region, climate, development time, and ovarian endocrine function [2]. At present, due to the general improvement in nutrition, health status, and living conditions, the occurrence of uterine fibroids should also be paid attention for young girls. The incidence of uterine fibroids increases rapidly after the age of 30 [3]. The prevalence of perimenopausal and menopausal uterine fibroids is not lower than that of premenopausal women.

During the past few decades, coordination polymers with intriguing topological architectures and high thermal stability have been widely explored as solid functional materials in the fields of luminescence sensing, catalysis, magnetism, molecular recognition, and so on [4–7]. In this context, numerous coordination polymers have been successfully synthesized *via* the self-assembly of various multidentate organic ligands and metal ions/polynuclear metal clusters [8–11]. Nevertheless, how to realize the controllable syntheses of coordination polymers is still a perplexing problem to synthetic chemists. In order to achieve this goal, much effort has been devoted to develop efficient synthetic strategy, and a series of efficient methods, such as dual-ligand strategy, second building block subunit strategy, pillar-layer method, post-synthetic modification method, and so on, for the construction of coordination polymers have been established [12–15]. Among these strategies, dual-ligand strategy is the most commonly used method for the construction of coordination polymers with expected structures and properties [16–18]. The synergistic action of carboxylate ligand and N-donor ligand is beneficial to not only realize the structural diversities of coordination polymers but also regulate the functional properties of coordination polymers. According to the reports, it can be found that 5-substituted isophthalic acid ligands in combination with various N-donor auxiliary ligands show good coordination abilities to transition metal ions [19,20]. Considering these in mind, we selected two 5-substituted isophthalic acid ligands (5-methoxyisophthalic acid and 5-propoxyisophthalic acid) and two different flexible N-donor ligands (1,3-bis(2-methylimidazolyl)propane and 1,3-bis(imidazolyl)propane) to constructed two new coordination polymers, namely [Cd(5-meo-ip)(bmip)]_n_ (**1**) and [Zn(5-pro-ip)(bip)]_n_·2n(H_2_O) (**2**) (5-meo-H_2_ip = 5-methoxyisophthalic acid, 5-pro-H_2_ip = 5-propoxyisophthalic acid, bmip = 1,3-bis(2-methylimidazolyl)propane bip = 1,3-bis(imidazolyl)propane). In addition, we also investigate the luminescent properties of **1**–**2** at room temperature. Through a series of biological experiments, the assessment of their application values on the uterine fibroids combined with ultrasound therapy and the investigation of specific mechanisms were conducted.

## Experimental

### Materials and instrumentation

All starting materials except 5-subsitited isophthalic acid used in this work were commercially available from Aladdin company. The 5-subsitited isophthalic acid ligands were synthesized by referring the reported literature [21]. Elemental analyses (C, H, and N) were determined using an elemental Vario EL III analyzer. Powder X-ray diffraction (PXRD) analyses were recorded on a PANalytical X’Pert Pro powder diffractometer with Cu/Kα radiation (λ = 1.54056 Å) with a step size of 0.05°. Thermogravimetric analyses for **1**–**2** were performed on a NETSCHZ STA-449C thermoanalyzer with a heating rate of 10 °C/min under nitrogen atmosphere in the temperature range of 30–800°C. The luminescence spectra for **1**–**2** and free organic ligands were measured on an Edinburg FLS920 TCSPC fluorescence spectrophotometer at room temperature. Caspase-3 Activity Assay Kit (ab252897) and Caspase-8 Activity Assay Kit (ab219915) were purchased from the Abcam.

### Synthesis of [Cd(5-meo-ip)(bmip)]_n_ (1) and [Zn(5-pro-ip)(bip)]_n_·2n(H_2_O)(2)

A mixture of Cd(NO_3_)_2_·4H_2_O (0.2 mmol), 5-meo-H_2_ip (0.2 mmol), bmip (0.2 mmol), Na_2_CO_3_ (0.2 mmol) and deionized H_2_O (8 mL) was sealed into a 20 mL Teflon-lined stainless steel and heated at 120°C for 3 days. After cooling to room temperature slowly, colorless block crystals of **1** were obtained in 45% yield based on Cd(II) salts. Anal. Calcd. (%) for C_20_H_22_CdN_4_O_5_: C, 46.98; H, 4.31; N, 10.96. Found (%): C, 46.52; H, 4.28; N, 10.93.

A mixture of Zn(NO_3_)_2_·6H_2_O (0.2 mmol), 5-pro-H_2_ip (0.2 mmol), bip (0.2 mmol), Na_2_CO_3_ (0.2 mmol) and deionized H_2_O (8 mL) was sealed into a 20 mL Teflon-lined stainless steel and heated at 120°C for 3 days. After cooling to room temperature slowly, colorless block crystals of **2** were obtained in 42% yield based on Zn(II) salts. Anal. calcd. (%) for C_20_H_26_ZnN_4_O_7_: C, 48.06; H, 5.24; N, 11.21. Found (%): C, 48.48; H, 4.02; N, 11.27. It should be noted that the H content in the found values of elemental analysis is less than the value of the calculated one, which might be due to the partly loss of lattice water molecules in the elemental analysis tests.

### X-ray crystallography

The structural data of compounds **1**–**2** were collected a computer–controlled Oxford Xcalibu E diffractometer with graphite–monochromated Mo–*Kα* radiation (λ = 0.71073 Å) at T = 293(2) K, and their structures were solved by the dual direct method using *ShelxT* and refined with the full-matrix least square technique based on *F*^2^ using the *SHELXL*-2014 [22]. Crystallographic data and structural refinements for compounds **1–2** are summarized in Table 1. Selected bond lengths (Å) and angles (°) of compounds **1**–**2** are given in Table S1. The hydrogen bond parameters of compound **1** was listed in Table S2.

**Table 1.** Crystal data and structure refinements for compounds **1**–**2.**

Sample	1	2
Formula	C_20_H_22_CdN_4_O_5_	C_20_H_26_ZnN_4_O_7_
*Fw*	510.81	499.82
Crystal system	orthorhombic	monoclinic
Space group	*P*bca	*P*2_1_/n
*a* (Å)	14.8291(18)	7.5915(18)
*b* (Å)	16.3662(19)	16.968(4)
*c* (Å)	16.828(2)	17.665(4)
*α*(°)	90	90
*β*(°)	90	100.936(4)
*γ*(°)	90	90
Volume (Å^3^)	4084.0(8)	2234.1(9)
*Z*	8	4
Density (calculated)	1.662	1.486
Abs. coeff. (mm^−1^)	1.110	1.148
Total reflections	23820	17031
Unique reflections	4519	5005
Goodness of fit on *F^2^*	1.090	1.070
Final *R* indices [*I* > 2sigma(*I*^2^)]	*R* = 0.0189, *wR*_2_ = 0.0449	*R* = 0.0525, *wR*_2_ = 0.1676
*R* (all data)	*R* = 0.0201, *wR*_2_ = 0.0455	*R* = 0.0612, *wR*_2_ = 0.1752
CCDC	2078360	2078361

### Annexin V-FITC Apoptosis detection

In order to assess the two compounds’ treatment activity on the uterine fibroids combined with ultrasound therapy and the influence of the two compounds on the uterine fibroid cells apoptosis, the Annexin V-FITC Apoptosis detection was performed in this present research. This conduction was finished in accordance with the instructions’ guidance, which has been slightly modified. In a word, the cells of uterine fibroid in the stage of logical growth were harvested and then inoculated them into plates of 6 well (10^5^ cells/well). After incubation with 5%CO_2_ at 37°C for 12 h, the cells were treated with compound **1** or compound **2** at 5 mg/mL concentration. Afterward, the cells were collected and then cleaned; then, they were labeled with 5 μL PI and Annexin V-FITC reagent, and the absorbance of all the samples was determined via the flow cytometry. This experiment was conducted in triplicate.

### Caspase-3 and Caspase-8 Activity Assay Kit

The Caspase-3 and Caspase-8 Activity Assay Kit was used in this present research to evaluate the activation levels of the apoptotic signaling pathway by measuring the activation levels of the capase-3 and caspase-8 in the uterine fibroid cells after compounds treatment. All the preformation in this experiment was based on the instruction manual, which has been slightly modified. In a word, the uterine fibroid cells in the stage of logical growth were collected and then inoculated them into plates of 6 well (10^5^ cells/well). After incubation with 5%CO_2_ at 37°C for half a day, the cells were treated using compound **1** or compound **2** at 5 mg/mL concentration. After the treatment, the cells were collected and lysed. Ac-DEVD-pNA (2 mM) solution was added into the wells. All the samples were incubated at 37°C for 60–120 minutes, and the absorbance of all samples was measured at 405 nm. This conduction was repeated at least three times, and the results were presented as mean ± SD.

## Results and discussion

### Crystal structure of compound 1

**Figure 1.**
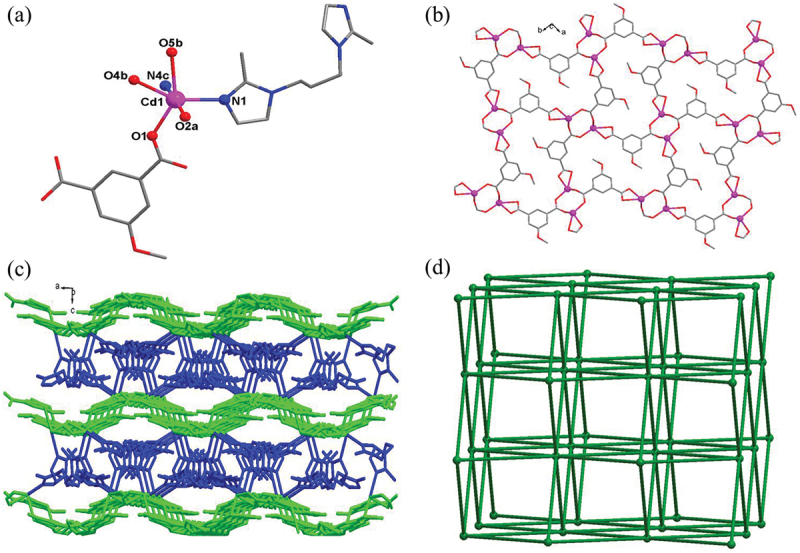
(a) Viewing of the coordination environment of Cd(II) ion in **1** (Pink: Cd, red: O, grey: C, blue: N). (b) The 2D layer constructed by 5-meo-ip^2-^ ligand and dinuclear [Cd_2_(COO)_2_] subunits. (c) The 3D framework of **1**. (d) The 8-connected bcu-type topological network for **1.**

Single crystal X-ray crystallography analysis characterized that the **1** crystallizes in orthorhombic *P*bca space group with the structure displaying a **bcu**-type topological network. The asymmetric unit of **1** is composed of one Cd(II) ion, one 5-meo-ip^2-^ ligand, as well as one bmip ligand. As shown in Figure 1(a), each Cd(II) ion displays a slightly distorted octahedral coordination geometry that was defined by four carboxylate oxygen atoms from three different 5-meo-ip^2-^ ligands, and two imidazolyl nitrogen atoms from two different bmip ligands. The Cd-O and Cd-N bond distances are in the range of 2.2390(12)-2.5285(11) Å, 2.2646(12)-2.3424(13) Å, respectively. The 5-meo-ip^2-^ ligand in **1** adopts a (*κ*^1^:*κ*^1^)-(*μ*_1_)-*μ*_3_ coordination mode connecting with three Cd(II) ions. Two adjacent Cd(II) ions with distances of 4.78 Å are bridged by two bis-monodentate carboxylate groups to give rise a dinuclear [Cd_2_(COO)_2_] subunit. These dinuclear [Cd_2_(COO)_2_] subunits are further connected together *via* the chelating carboxylate groups of 5-meo-ip^2-^ ligand, forming a 2D layer extending along crystallographical *ab* plane (Figure 1(b)). The bmip ligands adopt *cis-trans* conformation with the dihedral angle of 24.68° between two imidazole rings, which finally connected adjacent 2D layers into a 3D framework (Figure 1(c)). In this 3D framework, each 5-meo-ip^2-^ ligand and bmip ligand link two different dinuclear [Cd_2_(COO)_2_] subunits, and each dinuclear [Cd_2_(COO)_2_] subunit is coordinated by four 5-meo-ip^2-^ ligands and four bmip ligands. Therefore, the whole framework of **1** can be simplified into a 8-connected **bcu**-type topological network with the point symbol of {4^24^·6^4^} by viewing dinuclear [Cd_2_(COO)_2_] subunits as 8-connected nodes and organic ligands as linear connectors. (Figure 1(d)).

### Crystal structure of compound 2

**Figure 2.**
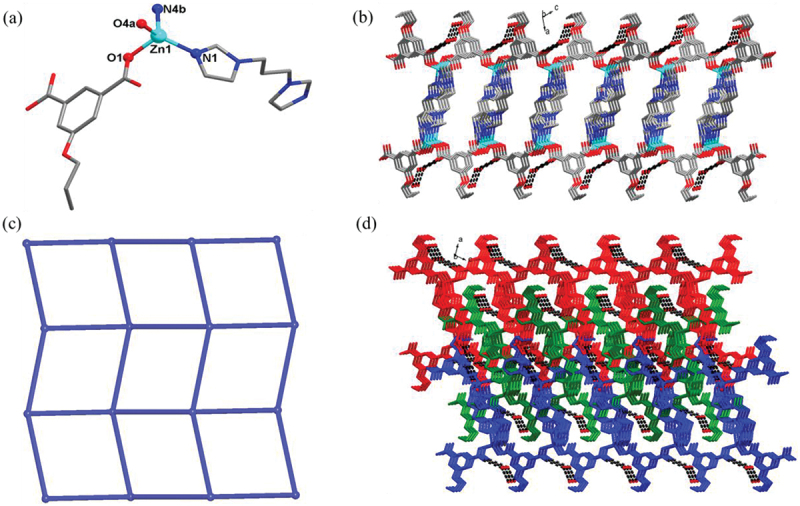
(a) Viewing of the coordination environments of Zn(II) ion in **2**. (b) The 2D layer structure of **2**. (c) The 4-connected **sql**-type tetragonal plane net for **2**. (d) The interdigitated 3D supramolecular framework of **2.**

X-ray structural characterization revealed that **2** crystallizes in monoclinic *P*2_1_/n space group and features a 2D layer structure with 4-connected **sql**-type tetragonal plane net. The asymmetric unit of **2** consists of one Zn(II) ions, one 5-pro-ip^2-^ ligand, one bip ligand, and two free water molecules. As shown in Figure 2(a), the Zn(II) ion locates in tetrahedral arrangement surrounded by two carboxylate oxygen atoms from two 5-pro-ip^2-^ ligands and two imidazolyl nitrogen atoms from two different bip ligands with the Zn-O and Zn-N bond distances in the range of 1.976(2)-2.015(2) Å, 2.027(2)-2.038(3) Å, respectively. In **2**, each 5-pro-ip^2-^ ligand links with two Zn(II) ions in (*κ*^1^:*κ*^0^)-(*κ*^1^:*κ*^0^)-*μ*_2_ mode, and each bip ligand adopts a *cis-cis* conformation with the dihedral angle of 80.49° between two imidazole rings. All Zn(II) ions are connected into a 2D layer structure via the bridging action of 5-pro-ip^2-^ ligands and bip ligands (Figure 2(b)). The lattice water molecules are connected with the 2D layer via the hydrogen bonds between O1w and O2w, O1w and carboxylate oxygen atom. By viewing Zn(II) ions and organic ligands as 4-connected nodes, linear connectors, respectively, this 2D layer of **2** can be simplified into a 4-connected **sql**-type tetragonal plane net with the point symbol of {4^4^·6^2^} (Figure 2(c)). Finally, viewing along crystallographic b direction, these 2D layers are interdigitated with each other under the weak Van der Waals forces, affording a 3D interdigitated supramolecular framework (Figure 2(d)).

### Powder X-ray diffraction patterns (PXRD) and thermogravimetric analyses (TGA)

The phase homogeneity of the obtained samples is demonstrated by the PXRD analyses of **1**–**2** at room temperature. As shown in Fig. S1, the experiment patterns of **1**–**2** are in good agreement with the corresponding simulated ones generated from the single crystal diffraction data, which indicates good phase purity and homogeneity of the bulk samples.

**Figure 3.**
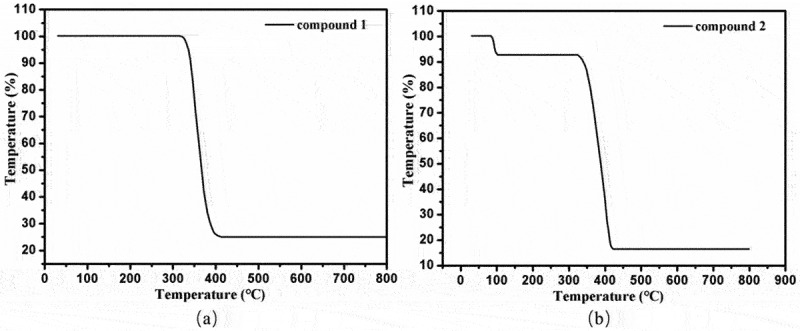
The TGA curves (a) for compound **1** and (b) for compound **2.**

The thermal stability of **1**–**2** was also characterized by the thermogravimetric analyses under nitrogen atmosphere, and the results of thermogravimetric analyses are shown in Figure 3. For compound **1**, the decomposition of framework began at 313°C, and ended at 412°C, leaving the final residues of 25.06% corresponding to the formation of CdO with the theory value of 25.14%. For compound **2**, the loss of lattice water molecules occurred in the temperature range of 82–102°C (obsd: 7.14%, calcd: 7.20%), and the decomposition of framework began at 322°C and ended at 424°C. The final residues of 16.44% may be ZnO (calcd: 16.21%).

### Luminescent properties of compounds 1-2

**Figure 4.**
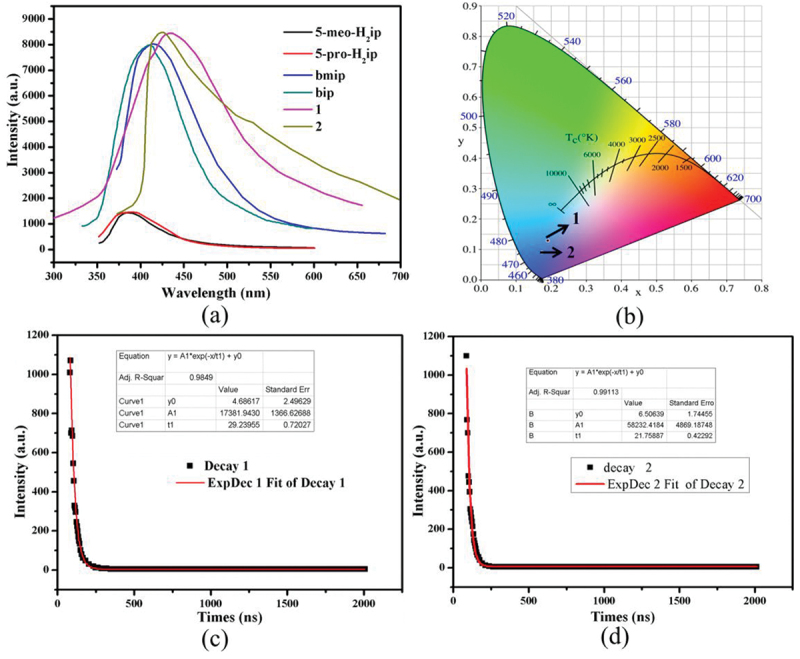
(a) The emission spectra of **1**–**2** and free organic ligands. (b) CIE-1931chromaticity diagram for **1**–**2**. (c) The luminescence lifetime decay curve for **1**. (d) The luminescence lifetime decay curve for **2.**

The excellent luminescent properties of d^10^ transition metal-based coordination polymers inspired us to measure the emission spectra of **1**–**2** at room temperature, and the results are displayed in Figure 4(a). Compounds **1**–**2** show intense luminescence with the emission bands centered at 432 nm (*λ*_ex_ = 340 nm), 424 nm (*λ*_ex_ = 340 nm), respectively. In order to understand the luminescent origin, the luminescent spectra of free organic ligands (5-meo-H_2_ip, 5-pro-H_2_ip, bmip and bip) were also measured at the same conditions. The emission bands for 5-meo-H_2_ip and 5-pro-H_2_ip are centered at 384 nm (*λ*_ex_ = 310 nm) and 386 nm (*λ*_ex_ = 310 nm), and the emission bands for bmip and bip are centered at 412 nm (*λ*_ex_ = 340 nm) and 408 nm (*λ*_ex_ = 340 nm). Based on previous literatures, these emissions of free organic ligands are mainly derived from intraligand π*→*π*/*n* electronic transitions [23]. Notably, the luminescence intensity of dicarboxylate ligands is much weaker than that of the bmip and bip ligands, indicating that the dicarboxylate ligands contribute little to the luminescence of compounds. Owing to d^10^ configurational Zn(II) and Cd(II) ions are difficult to reduce or oxidize, thus, the luminescence of compounds **1**–**2** can be tentatively assigned to bmip and bip intraligand transfer [24]. By calculation, it can be found that the CIE chromaticity coordinates of compounds **1**–**2** are at (0.1909, 0.1291), (0.1729, 0.0905), respectively, indicating that **1**–**2** can be served as excellent blue luminescence materials (Figure 4(b)). In addition, the luminescence lifetimes of **1**–**2** were also measured, and the calculated luminescence lifetimes are 29.2 ns for **1** and 21.8 ns for **2** (Figure 4(c,d)).

### Compound increased the levels of the uterine fibroid cells apoptosis

**Figure 5.**
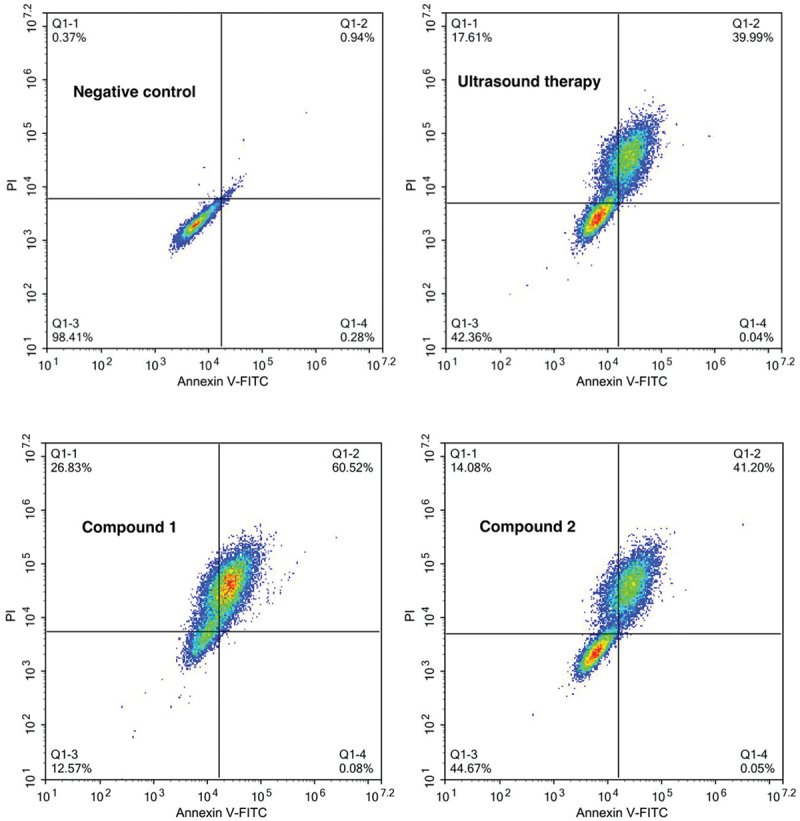
Increased uterine fibroid cells apoptosis levels after treated with the compound. The uterine fibroid cells were harvested and then inoculated into plates at the destiny of 10^5^ cells per well. Afterwards, the two compounds were added to conduct the treatment at 5 mg/mL concentration. The Annexin V-FITC Apoptosis detection was implemented for the determination of the uterine fibroid cells apoptosis levels.

After the synthesis of the two compounds, their treatment activity on the uterine fibroids combined with ultrasound therapy was first evaluated by the Annexin V-FITC Apoptosis detection by measuring the levels of the uterine fibroid cells apoptosis with flow cytometry. As the results showed in Figure 5, we can find that in comparison with negative control group, compound **1** could evidently increase the uterine fibroid cells apoptosis levels, but compound **2** only showed a slightly influence on the percentage of the uterine fibroid cells. This experiment indicated that compound **1** possesses better treatment effect by inducing the uterine fibroid cells apoptosis.

### Compound up-regulated the apoptotic signaling pathway activation in the uterine fibroid cells

**Figure 6.**
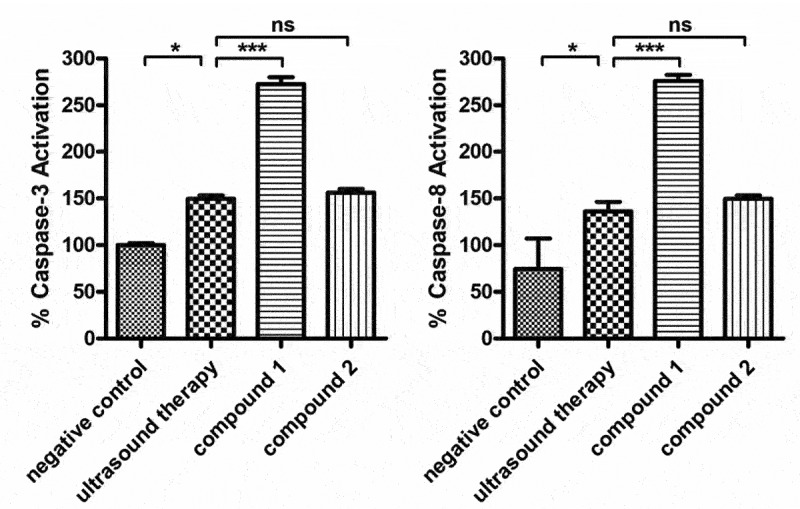
Up-regulated apoptotic signaling pathway in the uterine fibroid cells after treated with the compound. The uterine fibroid cells were harvested and then inoculated into plates at 10^5^ cells per well destiny. Afterwards, the two compounds were added to conduct the treatment with 5 mg/mL. The caspase-3 and capase-8 levels in the cells of uterine fibroid was detected via the caspase-3 (A) and caspase-8 (B) activity assay kit.

In former investigation, we have demonstrated that compound **1** exerted the outstanding treatment effects against uterine fibroids by inducing the uterine fibroid cells apoptosis. Furthermore, whether the compound could also influence the activation of the apoptotic signaling pathway was further explored in the present experimental study. The results in Figure 6 showed that compared with the control group, the ultrasound therapy could slightly increase the activation of caspase-3 and caspase-8, which could be obviously enhanced by compound **1**. Different from compound **1**, the biological activity of compound **2** was much weaker than that of compound **1**.

## Conclusions

In summary, we have successfully synthesized and characterized two new Zn(II)/Cd(II) coordination polymers. Compound **1** features a 3D framework and can be simplified into an 8-connected pcu-type topological net, and compound **2** features a 2D layer structure and can be reduced into 4-connected sql-tetragonal plane net. The luminescent emission spectra of **1**–**2** indicated that they can be served as excellent blue luminescence materials, and the luminescence lifetimes are 29.2 ns for **1** and 21.8 ns for **2**. The Annexin V-FITC Apoptosis detection showed compared with compound **2**, compound **1** has much stronger inhibitory activity. Besides, the expression levels of the capase-3 and caspase-8 was also up-regulated by compound **1**, which was much more excellent than compound **2**.

## Supplementary Material

Supplemental Material

## Data Availability

Selected bond lengths (Å) and angles (^°^) for compounds **1-2** (Table S1), the detailed hydrogen-bond parameters for compound **2** (Table S2), the PXRD patterns (a) for compound **1** and (b) for compound **2** (Fig. S1), the information could be found in the supporting information file.
