# Supplementary material for: Two new Cd(II)/Zn(II) coordination polymers: luminescence properties and synergistic treatment activity with ultrasound therapy on uterine fibroids
Source: Des Monomers Polym. 2022 Jun 20;25(1):197–204. doi: 10.1080/15685551.2022.2088976 (PMC9225708; doi:10.1080/15685551.2022.2088976)
Supplement: Supplemental Material [file TDMP_A_2088976_SM4597.doc]

**Table S1 Selected bond lengths (Å) and angles (°) for compounds 1-2.**

| **Compound 1** |  |  |  |
| --- | --- | --- | --- |
| Cd(1)-O(1) | 2.2390(12) | Cd(1)-O(2)^1^ | 2.3198(10) |
| Cd(1)-O(4)^2^ | 2.2945(10) | Cd(1)-O(5)^2^ | 2.5285(11) |
| Cd(1)-N(1) | 2.2646(12) | Cd(1)-N(4)^3^ | 2.3424(13) |
| O(1)-Cd(1)-O(2)^1^ | 93.22(4) | O(1)-Cd(1)-O(4)^2^ | 94.87(4) |
| O(1)-Cd(1)-O(5)^2^ | 147.70(4) | O(1)-Cd(1)-N(1) | 113.48(4) |
| O(1)-Cd(1)-N(4)^3^ | 82.47(4) | O(2)^1^-Cd(1)-O(5)^2^ | 96.03(4) |
| O(2)^1^-Cd(1)-N(4)^3^ | 137.39(6) | O(4)^2^-Cd(1)-O(2)^1^ | 89.05(4) |
| O(4)^2^-Cd(1)-O(5)^2^ | 54.55(4) | O(4)^2^-Cd(1)-N(4)^3^ | 93.12(4) |
| N(1) -Cd(1)-O(2)^1^ | 89.63(4) | N(1) -Cd(1)-O(4)^2^ | 151.66(4) |
| N(1) -Cd(1)-O(5)^2^ | 97.49(4) | N(1) -Cd(1)-N(4)^3^ | 90.42(5) |
| N(4)^3^-Cd(1)-O(5)^2^ | 88.61(4) |  |  |
| **Compound 2** |  |  |  |
| Zn(1)-O(1) | 1.976(2) | Zn(1)-O(4)^1^ | 2.015(2) |
| Zn(1)-N(1) | 2.027(2) | Zn(1)-N(4)^2^ | 2.038(3) |
| O(1)-Zn(1)-O(4)^1^ | 101.47(9) | O(1)-Zn(1)-N(1) | 108.83(9) |
| O(1)-Zn(1)-N(4)^2^ | 97.76(10) | O(4)^1^-Zn(1)-N(1) | 128.36(9) |
| O(4)^1^-Zn(1)-N(4)^2^ | 111.88(10) | N(1)-Zn(1)-N(4)^2^ | 104.37(10) |

Symmetry codes: compound **1**: (1) 1 – *x*,1 – *y*, 1 – *z*; (2)1/2 – *x*, –1/2 + *y*, *z*; (3) 1 – *x*, 1/2 + *y*, 3/2 – *z*. compound **2**: (1) –1/2 + *x*, 3/2 – *y*, –1/2 + *z*; (2) –1/2 – *x*, 1/2 + *y*, 1/2 – *z*.

**Table S2 The detailed hydrogen-bond parameters for compound 2.**

| D−H…A | D−H | H…A | D…A | ∠ D−H…A |
| --- | --- | --- | --- | --- |
| O1w-H1wa-O2 | 0.85 | 2.16 | 2.926 | 150 |
| O2w-H2wa-O1w | 0.85 | 2.17 | 2.934 | 149 |


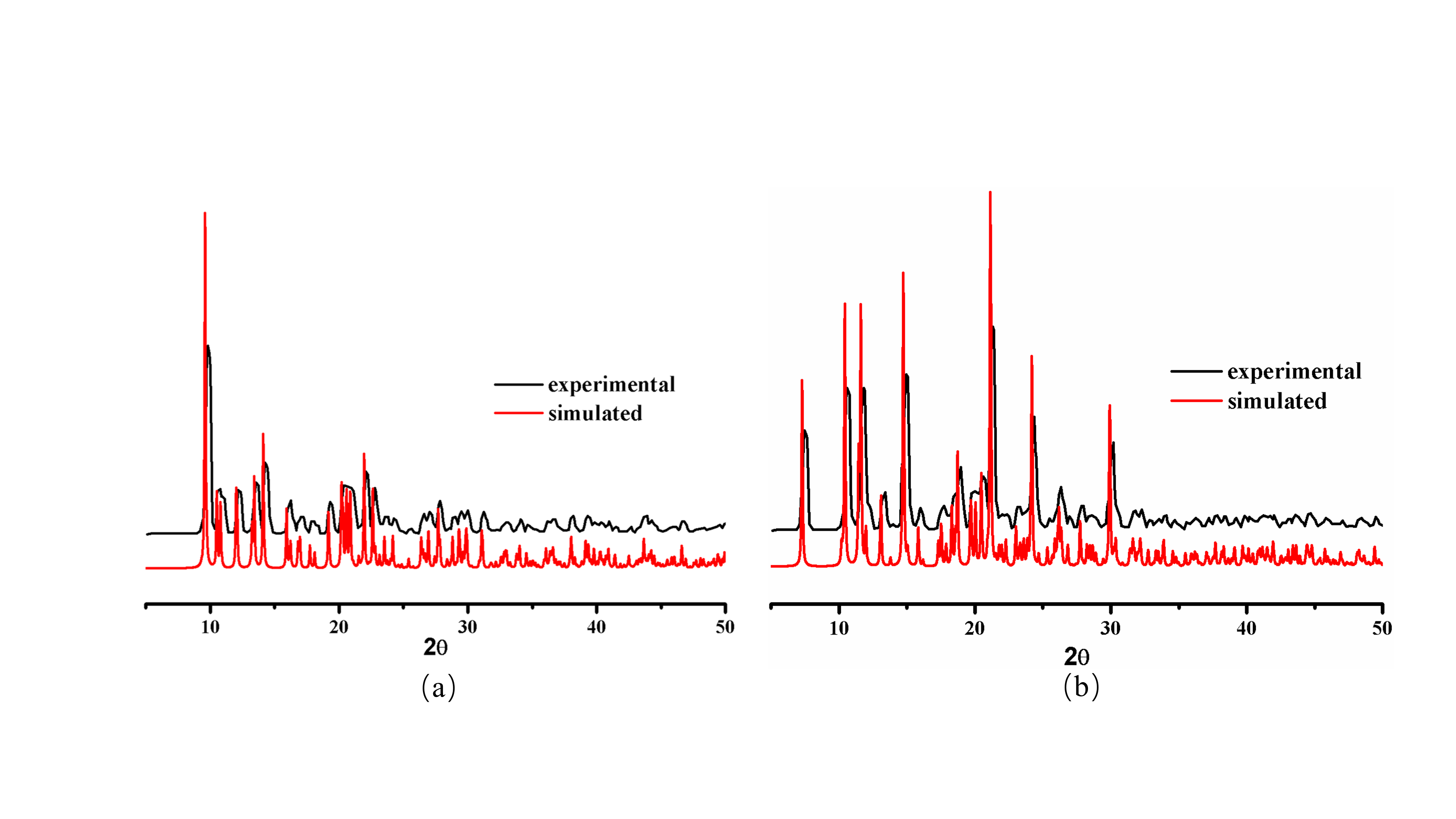


**Fig.S1.** The PXRD patterns (a) for compound **1** and (b) for compound **2**.
